# Endoscopic Ultrasound-Guided Oncologic Therapy for Pancreatic Cancer

**DOI:** 10.1155/2013/157581

**Published:** 2013-02-24

**Authors:** Rei Suzuki, Atsushi Irisawa, Manoop S. Bhutani

**Affiliations:** Department of Gastroenterology, Hepatology and Nutrition, The University of Texas MD Anderson Cancer Center, Unit 1466, 1515 Holcombe Bovlevard, Houston, TX 77030-4009, USA

## Abstract

Since the development of endoscopic ultrasound-guided fine needle aspiration (EUS-FNA) in the early 1990s, its application has been extended to various diseases. For pancreatic cancer, EUS-FNA can obtain specimens from the tumor itself with fewer complications than other methods. Interventional EUS enables various therapeutic options: local ablation, brachytherapy, placement of fiducial markers for radiotherapy, and direct injection of antitumor agents into cancer. This paper will focus on EUS-guided oncologic therapy for pancreatic cancer.

## 1. Introduction

Since endoscopic ultrasound-guided fine needle aspiration (EUS-FNA) was first utilized in clinical practice in the early 1990s, it has become a widely acceptable diagnostic procedure in gastrointestinal and pancreaticobiliary lesions [1]. Recently, its applications have been extended to therapeutic purposes for various diseases, including pancreatic cancer [2–4].

Pancreatic ductal adenocarcinoma is one of the most difficult tumors to treat, and patients with this disease have a poor prognosis. Only 15 to 20% of patients have resectable disease at diagnosis; approximately 40% have metastatic disease, and another 30 to 40% have locally advanced unresectable tumors. Despite the tremendous efforts of researchers and clinicians, the 5-year survival of patients with unresectable pancreatic cancer is still <5% [5]. To improve this dismal prognosis, endoscopic therapy (local therapy alone or in combination with systemic therapy) is considered as one option for such advanced cancers. The conventional approach is percutaneous image-guided delivery, for example, computed tomography (CT) guided, but this technique is cumbersome. EUS may be a suitable procedure for approaching the cancer itself via the gastrointestinal tract under real-time color Doppler, thus avoiding blood vessels or other organs. In this paper, we consider a variety of EUS-guided oncologic therapies for pancreatic cancer. 

## 2. EUS-Guided Ablation

Even though pancreatic cancer is considered to be a systemic disease, local ablation therapy has the potential to reduce tumor volume, thereby improving patients' symptoms and prognosis. 

### 2.1. Photodynamic Therapy

Photodynamic therapy (PDT) provides localized tissue ablation through the use of an appropriate photosensitizer and light exposure. Photosensitizers (e.g., *meso*-tetra(hydroxyphenyl)chlorin, porfimer sodium, and verteporfin), which have unique characteristics and accumulate more in tumors than in normal tissue, are usually injected intravenously before the procedure. Light is generated with small optic fibers that can go through a 19-gauge needle. The first PDT for gastrointestinal disease was performed in patients with dysplasia arising from Barrett's esophagus [6, 7]. Later, Bown et al. performed CT-guided percutaneous PDT therapy in 16 patients with unresectable pancreatic cancer [8] and observed localized necrosis without pancreatitis. Subsequently, EUS-guided PDT was performed by Chan et al. [9] and Yusuf et al. [10] in normal animal pancreata. Their studies found localized pancreatic necrosis without significant complications.

### 2.2. Radiofrequency Ablation

Radiofrequency (RF) ablation is a widely accepted therapeutic procedure for unresectable liver tumors. For pancreatic cancer, the conventional approach techniques are transabdominal (ultrasound- or CT-guided) or laparoscopic. In 1999, Goldberg et al. first reported the use of EUS-guided RF in an animal model [11]. Normal pig pancreas was localized with EUS and a 19-gauge needle electrode was introduced via the stomach. Hyperlipasemia and focal pancreatitis, and later pancreatic fluid collection, were observed in only one pig. At autopsy, localized ablation was observed in all 13 pigs. Carrara et al. developed a newly designed gas-cooled RF ablation probe and performed RF in 14 normal pig pancreata [12, 13]. This 1.8 mm bipolar probe with a sharp distal tip was internally cooled with carbon dioxide, allowing efficient cooling owing to the Joule-Thomson effect. They reported that this new probe created an ablation zone with the extent depending on the duration of the RF application and with fewer complications than other RF ablation techniques. 

### 2.3. Nd : YAG Laser and High-Intensity Focused Ultrasound

The application of the neodymium-doped yttrium aluminum garnet (Nd : YAG) laser for pancreatic diseases has been limited. Di Matteo et al. performed EUS-guided Nd : YAG laser ablation in a pig model [14]. In this study, a well-demarcated necrosis was observed with no severe complications. Animal studies and a few clinical trials have suggested the potential benefits of EUS-guided ablation for pancreatic cancer. 

 High-intensity focused ultrasound (HIFU) is a rapidly developing technology that is widely used for noninvasive ablation of tumors [15, 16]. Hwang et al. developed new-designed HIFU transducer which attached to EUS scope. They successfully could target and create ablation in porcine pancreas and liver [17].

 However, further studies are required to determine safety and technical feasibility of these techniques.

## 3. EUS-Guided Brachytherapy and EUS-Guided Fiducial Markers 

Radiation therapy is considered an option for controlling locally advanced pancreatic cancer. Conventionally, fractional external beam radiation therapy with chemotherapy has been used for such patients. Other emerging therapeutic options are interstitial brachytherapy and image-guided radiotherapy. 

Interstitial brachytherapy has been used to control malignancies of the prostate, breast, and brain. The technique has been also considered to have potential benefit for pancreatic cancer and lymph node metastasis [18, 19]. Radioactive I-125 seeds are implanted during laparotomy or by a percutaneous approach. These seeds generate gamma rays and damage tumor tissue from inside. The EUS-guided approach is an alternative that allows precise and close visualization of the gastrointestinal tract and structures in the abdominal cavity, including the pancreas. 

Sun et al. first reported on EUS-guided brachytherapy in a porcine model using six pigs [20]. They implanted I-125 seeds (4.5 mm long and 0.85 mm thick) into normal pig pancreas under EUS guidance. The procedure was successful in all pigs and no migration of seeds occurred. On day 7, repeat EUS demonstrated heterogenous hypoechoic lesions surrounding seeds in all pigs. The median diameter of the lesions was 32 mm (range 28–35 mm) and increased to 38 mm on day 14. Only one pig had Hyperlipasemia. Autopsy showed 35 to 45 mm localized necrosis surrounded by fibrotic tissue with mild inflammation. This same group later conducted a pilot study in 15 patients with locally advanced pancreatic cancer (eight patients with stage III, seven with stage IV). EUS-guided brachytherapy was used alone, without external irradiation or any chemotherapy. Hematological toxicity was tolerable but included grade III neutropenia, anemia, and thrombocytopenia. Pancreatitis occurred in three patients. This treatment showed some clinical benefit in 30% of the patients. Overall median survival was 10.6 months (range from 4.2 to 25 months). The results of this study were similar to the findings of a study by Jin et al., which combined EUS-guided brachytherapy with gemcitabine and 5-fluorouracil (5-FU) chemotherapy. In this latter study, researchers implanted I-125 seeds into 22 patients depending on the tumor volume (median 10 seeds, range from 5 to 30) [21]. One week after the initial implantation, patients began a 5-day intravenous schedule of gemcitabine (1.0 g/m^2^) on the first day followed by 4 days of 5-FU. This regimen was repeated every 4 weeks up to six cycles if tolerated. Although the tumor growth was effectively controlled (stable disease, partial remission) in 59.1% of patients and pain was relieved in all patients, median survival time was 9.0 months, indicating no significant improvement compared with previously published metadata [22]. 

In conclusion, EUS-guided brachytherapy has shown safety and partial benefits in animal and clinical pilot studies. Further studies are required to determine its clinical application.  Randomized  clinical  trials  (e.g.,  brachytherapyalone  versus  brachytherapy/chemotherapy or brachytherapy/external radiotherapy versus brachytherapy/external radiotherapy + chemotherapy) may lead to new conclusions.

Image-guided radiotherapy includes various types of radiotherapy. Recently, stereotactic body radiotherapy (SBRT) and intensity-modulated radiotherapy (IMRT) have been considered as less toxic than conventional external fractionated radiotherapy because of their precise and pinpoint accuracy. Clinical application of these techniques was previously limited to intracranial tumors because they required a stereotactic frame, but the development of the CyberKnife frameless radiosurgery system (Accuray, Sunnyvale, CA; USA) changed the practice. Nonintracranial tumors can be treated with implantable radiographic markers (or fiducials) as reference points. Conventional techniques to place fiducial markers are surgery or a percutaneous approach. EUS guidance is also feasible here.

The first report of EUS-guided fiducial marker placement was published in 2006 by Pishvaian et al., who successfully placed fiducial markers in six of seven pancreatic cancer patients, with no observed complications [23]. Later, Varadarajulu et al. confirmed the feasibility and safety of EUS-guided fiducial marker placement to deliver IMRT [24].

However, the role of such radiotherapies (SBRT and IMRT) for pancreatic cancer is still unclear. If randomized control trials can demonstrate the superiority of such techniques over conventional external fractionated radiotherapy, the clinical value of EUS-guided fiducial marker placement will be clear. 

## 4. EUS-Guided Antitumor Agent Injection

Endosonographers have tried various agents, including oncolytic virus, mixed lymphocyte culture, and immature dendritic cells, for the treatment for pancreatic cancer patients. We here summarize the results of published studies that have considered EUS-guided antitumor agent injection.

### 4.1. Allogenic Mixed Lymphocyte Culture (Cytoimplant)

The results of the first EUS-guided antitumor agent injection study were published in 2000 by Chang et al. [25]. They utilized allogeneic mixed lymphocyte culture (cytoimplant). Cytoimplant was generated by coincubation of host and allogeneic donor peripheral blood mononuclear cells and resulted in the release of cytokines and the activation of immune effector cells. These conjugates were injected into pancreatic cancer locally under EUS guidance in eight patients in a dose-escalating manner (3, 6, and 9 billion cells). There were no procedure-related complications. There were two partial responses and one minor response, with a median survival of 13.2 months. Currently, there are no active clinical protocols evaluating cytoimplant for pancreatic cancer.

### 4.2. Immature Dendritic Cells

Dendritic cells (DCs) are potent antigen-presenting cells that can stimulate naïve T lymphocytes to develop into tumor-specific cytolytic cells. Immunotherapy studies using these cells have been published for melanoma, kidney, and prostate cancers. The first results for an animal model of pancreatic cancer were published in 2003 by Akiyama et al., who found tumor growth inhibition in 82% of mice [26]. Later, Irisawa et al. performed EUS-guided injection of immature DCs in seven patients with prior chemotherapy (e.g., gemcitabine) failure [27]. Immature DCs were generated by the patients' peripheral blood mononuclear cells after stimulation with granulocyte-macrophage colony-stimulating factor (GM-CSF) and interleukin-4. Five of the seven patients underwent irradiation prior to the initial DC injection to facilitate tumor-associated antigens for DC cross-presentation. All DC injections were tolerated, with no clinical toxicity. No complications associated with the procedure were noted, and median patient survival was 9.9 months. Hirooka et al. utilized OK-432-pulsed DCs and mature DCs combined with gemcitabine for five patients with unresectable pancreatic cancer [28]. They postulated that OK-432-pulsed DCs would produce more antitumor effect that nonpulsed DCs based on a previous study on glioma [29]. Mature DCs and gemcitabine were used as the initial treatment in their study. They had promising results, with a median patient survival time of 15.9 months. 

### 4.3. Oncolytic Virus Therapy (ONYX-015)

Many types of oncolytic viruses have been studied as therapeutic agents for pancreatic cancer. Oncolytic viruses utilized in experimental and clinical works include adenoviruses, herpesviruses, and reoviruses.

The oncolytic effect has several possible mechanisms. The first such mechanism is the result of viral replication within the cell and rupture out of the cell. This cycle is repeated with progeny virions infecting and replicating in neighboring cells. The second mechanism involves a few viruses producing a cytotoxic protein during replication in cells, with the protein itself then causing damage to the cell. For example, late in the cell cycle, adenoviruses express a protein that is toxic to cells. The third mechanism involves virus infection of cancer cells, which induces antitumoral immunity. Basically, the cancer cell antigen is a weak antigen for host immune sensitization. The virus infiltration causes inflammation and lymphocyte penetration into the tumor, and the virus protein leads to increased sensitivity to tumor necrosis factor-mediated killing. To date, ONYK-015 given by EUS-guided injection has been used for pancreatic patients in clinical studies. 

ONYK-015 is an E1B-55 kDa gene-deleted, replicated-selective adenovirus that preferentially replicates in and kills malignant cells. CT-guided ONYX-015 intratumoral injection for pancreatic cancer was performed by Mulvihill et al., who found a minor response in six of 22 patients [30]. However, the CT-guided approach is troublesome and difficult to perform repeatedly during a given treatment session. Considering the histological nature of pancreatic cancer with abundant fibrosis and normal tissue, repeat intratumoral injection would spread viral agents diffusely throughout the tumor. Hecht et al. performed EUS-guided ONYX-015 injection with intravenous gemcitabine in 21 patients with unresectable pancreatic cancers, with a median survival time of 7.5 months [31]. There were no procedure-related complications. 

### 4.4. TNFerade

TNFerade is a novel means, using gene transfer, of selective delivery of TNF-*α* to tumor cells. TNFerade consists of a portion of the radiation-inducible Egr-1 promoter gene upstream to a TNF-*α*  cDNA and incorporates this into an E1, E4, and E3, replication-deficient adenovirus type 5, providing spatial and temporal control of the radiosensitivity and cytotoxicity provided by TNF-*α* [32]. A phase I clinical study with percutaneous TNFerade injection combined with adjunctive radiation therapy demonstrated an objective response in patients with various solid tumors, including four pancreatic cancers [33]. Hecht et al. reported the results of a phase I/II study to evaluate the efficacy of TNFerade combined with chemoradiotherapy. TNFerade (at 4 × 10^9^, 4 × 10^10^, 4 × 10^11^, and 1 × 10^12^ particle units in 2 mL) was delivered by a percutaneous approach or via EUS guidance, with intravenous 5-FU (200 mg/m^2^ daily, 5 days/week) and radiation (50.4 Gy) [34]. Fifty patients were enrolled in this multicenter study, and the long-term follow-up results showed that the toxicity related to TNFerade was mild and tolerated in the lower dose groups (4 × 10^9^, 4 × 10^10^, and 4 × 10^11^ particle units). However, three of the nine patients assigned to the 10^12^ particle unit group had TNFerade-related toxicities (two instances of pancreatitis, one of cholangitis). Thus, the maximum tolerated dose was set at 4 × 10^11^ particle units. Additionally, such patients receiving 4 × 10^11^ particle units had a longer median survival (332 days) than the other groups (overall median survival was 297 days). There were no differences in overall outcome between the percutaneous (*n* = 23) and the EUS-guided (*n* = 27) approaches. A recent phase III trial that compared 5-FU chemoradiation therapy with or without TFNerade failed to show efficacy, and there are no active clinical protocols evaluating TNFerade for pancreatic cancer.

## 5. Conclusions

EUS-guided oncological therapy for pancreatic cancer has been studied in animal models and clinical trials. Some of these studies have shown positive effects in pancreatic cancer. Endosonographers can play an important role for patients with pancreatic cancer by delivering antitumor agents, performing ablation, and helping in radiotherapy.

## References

[1] Vilmann P, Jacobsen GK, Henriksen FW, Hancke S (1992). Endoscopic ultrasonography with guided fine needle aspiration biopsy in pancreatic disease. *Gastrointestinal Endoscopy*.

[2] Chang KJ, Irisawa A (2009). EUS 2008 Working Group document: evaluation of EUS-guided injection therapy for tumors. *Gastrointestinal Endoscopy*.

[3] Seo DW (2010). EUS-guided antitumor therapy for pancreatic tumors. *Gut and Liver*.

[4] Hara K, Yamao K, Mizuno N (2011). Interventional endoscopic ultrasonography for pancreatic cancer. *World Journal of Clinical Oncology*.

[5] Sener SF, Fremgen A, Menck HR, Winchester DP (1999). Pancreatic cancer: a report of treatment and survival trends for 100,313 patients diagnosed from 1985–1995, using the National Cancer Database. *Journal of the American College of Surgeons*.

[6] Regula J, MacRobert AJ, Gorchein A (1995). Photosensitisation and photodynamic therapy of oesophageal, duodenal, and colorectal tumours using 5 aminolaevulinic acid induced protoporphyrin IX—a pilot study. *Gut*.

[7] Overholt BF, Panjehpour M, Tefftellar E, Rose M (1993). Photodynamic therapy for treatment of early adenocarcinoma in Barrett’s esophagus. *Gastrointestinal Endoscopy*.

[8] Bown SG, Rogowska AZ, Whitelaw DE (2002). Photodynamic therapy for cancer of the pancreas. *Gut*.

[9] Chan HH, Nishioka NS, Mino M (2004). EUS-guided photodynamic therapy of the pancreas: a pilot study. *Gastrointestinal Endoscopy*.

[10] Yusuf TE, Matthes K, Brugge WR (2008). EUS-guided photodynamic therapy with verteporfin for ablation of normal pancreatic tissue: a pilot study in a porcine model (with video). *Gastrointestinal Endoscopy*.

[11] Goldberg SN, Mallery S, Gazelle GS, Brugge WR (1999). EUS-guided radiofrequency ablation in the pancreas: results in a porcine model. *Gastrointestinal Endoscopy*.

[12] Carrara S, Arcidiacono PG, Albarello L (2008). Endoscopic ultrasound-guided application of a new hybrid cryotherm probe in porcine pancreas: a preliminary study. *Endoscopy*.

[13] Carrara S, Arcidiacono PG, Albarello L (2008). Endoscopic ultrasound-guided application of a new internally gas-cooled radiofrequency ablation probe in the liver and spleen of an animal model: a preliminary study. *Endoscopy*.

[14] Di Matteo F, Martino M, Rea R (2010). EUS-guided Nd:YAG laser ablation of normal pancreatic tissue: a pilot study in a pig model. *Gastrointestinal Endoscopy*.

[15] Kim J, Chung DJ, Jung SE, Cho SH, Hahn S-T, Lee JM (2012). Therapeutic effect of high-intensity focused ultrasound combined with transarterial chemoembolisation for hepatocellular carcinoma <5 cm: comparison with transarterial chemoembolisation monotherapy—preliminary observations. *British Journal of Radiology*.

[16] Leslie T, Ritchie R, Illing R (2012). High-intensity focused ultrasound treatment of liver tumours: post-treatment MRI correlates well with intra-operative estimates of treatment volume. *British Journal of Radiology*.

[17] Hwang J, Farr N, Morrison K (2011). Development of ana EUS-guided high-intensity focused ultrasound endoscope. *Gastrointestinal Endoscopy*.

[18] Zhongmin W, Yu L, Fenju L, Kemin C, Gang H (2010). Clinical efficacy of CT-guided iodine-125 seed implantation therapy in patients with advanced pancreatic cancer. *European Radiology*.

[19] Kishi K, Sonomura T, Shirai S (2011). Brachytherapy reirradiation with hyaluronate gel injection of paraaortic lymphnode metastasis of pancreatic cancer: paravertebral approach—a technical report with a case. *Journal of Radiation Research*.

[20] Sun S, Qingjie L, Qiyong G, Mengchun W, Bo Q, Hong X (2005). EUS-guided interstitial brachytherapy of the pancreas: a feasibility study. *Gastrointestinal Endoscopy*.

[21] Jin Z, Du Y, Li Z, Jiang Y, Chen J, Liu Y (2008). Endoscopic ultrasonography-guided interstitial implantation of iodine 125-seeds combined with chemotherapy in the treatment of unresectable pancreatic carcinoma: a prospective pilot study. *Endoscopy*.

[22] Xie DR, Liang HL, Wang Y, Guo SS, Yang Q (2006). Meta-analysis on inoperable pancreatic cancer: a comparison between gemcitabine-based combination therapy and gemcitabine alone. *World Journal of Gastroenterology*.

[23] Pishvaian AC, Collins B, Gagnon G, Ahlawat S, Haddad NG (2006). EUS-guided fiducial placement for CyberKnife radiotherapy of mediastinal and abdominal malignancies. *Gastrointestinal Endoscopy*.

[24] Varadarajulu S, Trevino JM, Shen S, Jacob R (2010). The use of endoscopic ultrasound-guided gold markers in image-guided radiation therapy of pancreatic cancers: a case series. *Endoscopy*.

[25] Chang KJ, Nguyen PT, Thompson JA (2000). Phase I clinical trial of allogeneic mixed lymphocyte culture (cytoimplant) delivered by endoscopic ultrasound—guided fine-needle injection in patients with advanced pancreatic carcinoma. *Cancer*.

[26] Akiyama Y, Maruyama K, Nara N (2002). Antitumor effects induced by dendritic cell-based immunotherapy against established pancreatic cancer in hamsters. *Cancer Letters*.

[27] Irisawa A, Takagi T, Kanazawa M (2007). Endoscopic ultrasound-guided fine-needle injection of immature dendritic cells into advanced pancreatic cancer refractory to gemcitabine: a pilot study. *Pancreas*.

[28] Hirooka Y, Itoh A, Kawashima H (2009). A combination therapy of gemcitabine with immunotherapy for patients with inoperable locally advanced pancreatic cancer. *Pancreas*.

[29] Yu JS, Liu G, Ying H, Yong WH, Black KL, Wheeler CJ (2004). Vaccination with tumor lysate-pulsed dendritic cells elicits antigen-specific, cytotoxic T-cells in patients with malignant glioma. *Cancer Research*.

[30] Mulvihill S, Warren R, Venook A (2001). Safety and feasibility of injection with an E1B-55 kDa gene-deleted, replication-selective adenovirus (ONYX-015) into primary carcinomas of the pancreas: a phase I trial. *Gene Therapy*.

[31] Hecht JR, Bedford R, Abbruzzese JL (2003). A phase I/II trial of intratumoral endoscopic ultrasound injection of ONYX-015 with intravenous gemcitabine in unresectable pancreatic carcinoma. *Clinical Cancer Research*.

[32] Sharma A, Mani S, Hanna N (2001). An open-label, phase I, dose-escalation study of tumor necrosis factor-*α* (TNFerade biologic) gene transfer with radiation therapy for locally advanced, recurrent, or metastatic solid tumors. *Human Gene Therapy*.

[33] Senzer N, Mani S, Rosemurgy A (2004). TNFerade biologic, an adenovector with a radiation-inducible promoter, carrying the human tumor necrosis factor alpha gene: a phase I study in patients with solid tumors. *Journal of Clinical Oncology*.

[34] Hecht JR, Farrell JJ, Senzer N (2012). EUS or percutaneously guided intratumoral TNFerade biologic with 5-fluorouracil and radiotherapy for first-line treatment of locally advanced pancreatic cancer: a phase I/II study. *Gastrointestinal Endoscopy*.

